# The versatility of a glycerol-preserved skin allograft as an adjunctive treatment to free flap reconstruction

**DOI:** 10.4103/0970-0358.53017

**Published:** 2009

**Authors:** A. Z. Mat Saad, T. L. Khoo, A. A. Dorai, A. S. Halim

**Affiliations:** Department of Reconstructive Sciences, Hospital Universiti Sains Malaysia, 16150 Kubang Kerian, Kelantan, Malaysia

**Keywords:** Free flap, Free tissue transfer, Skin allograft

## Abstract

Skin allografts have been used in medical practice for over a century owing to their unique composition as a biological dressing. Skin allografts can be obtained in several preparations such as cryopreserved, glycerol-preserved, and fresh allograft. A glycerol-preserved allograft (GPA) was introduced in the early 1980s. It has several advantages compared with other dressings such as ease of processing, storage and transport, lower cost, less antigenicity, antimicrobial properties, and neo-vascularisation promoting properties. Skin allografts are mainly used in the management of severe burn injuries, chronic ulcers, and complex, traumatic wounds. Published reports of the use of skin allografts in association with free flap surgery are few or non existent. We would like to share our experience of several cases of free tissue transfer that utilised GPA as a temporary wound dressing in multiple scenarios. On the basis of this case series, we would like to recommend that a GPA be used as a temporary dressing in conjunction with free flap surgery when required to protect the flap pedicle, allowing time for the edema to subside and the wound can then be closed for a better aesthetic outcome.

## INTRODUCTION

Skin allografts have been used in medical practice for more than a century and are available in several forms. A fresh skin allograft is believed to be the best,[1] but it has limited resources and can be difficult to obtain. Cryopreserved and glycerol-preserved allografts (GPA) are other forms of skin allografts that are harvested, processed, and can be stored for a long time. This makes them more readily available compared with fresh skin allografts.

The benefits of skin allografts as a biological dressing are well known through its contribution to burn management for many decades. These include protection of the wound bed against dessication, colonization, heat, electrolytes, and protein loss by creating a physiological as well as mechanical barrier. Fresh cadaver allografts are still considered to be the gold standard skin substitute by several authors[1,2] but their scarce availability has severely impeded their use. The advantages of GPA compared with cryopreserved and fresh allograft preparations are enhanced revascularization of the wound bed by promoting neo-vascularisation, low antigenicity resulting in a decreased rejection reaction,[2,3] marked shortening of healing time,[2–4] enhanced wound epithelialisation, prevention of wound bed deterioration and secondary necrosis,[3,5] reduction in the risk of transmissible disease,[1] and many more that have been documented and reported.[1–7]

The low antigenicity of the GPA is due to its preparation with glycerol, which removes the vital components of the cell and renders the cell non viable.[1,8] A previous study has shown that the rejection of GPA is due to the inflammatory processes that is most probably mediated by host monocyte infiltration rather than by a rejection process mediated by T-cells.[9] Meanwhile, GPA has been demonstrated to have a lower risk of transmissible disease, even with HIV due to its preservation process. This depends on its glycerol concentration, time, and temperature exposure.[1,10,11]

Reconstruction following oncological surgery and trauma may require a free tissue transfer. Sometimes, these procedures can be prolonged and associated with tissue edema and swelling, preventing tension-free wound closure. This can cause compression or pressure to the flap pedicle thus compromising the flap perfusion. When available, GPA can be used temporarily as a biological dressing to cover the critical area while buying time to allow the edema and swelling to subside and vascular anastomosis to stabilise prior to secondary suturing or definitive wound closure with split thickness skin graft (SSG).

We would like to highlight the advantages of using skin allograft in different scenarios outside the scope of burn injury in three illustrated cases of reconstruction following oncological resections and a traumatic case using free tissue transfers.

## CASE REPORTS

### Case 1

**Figure 1:**
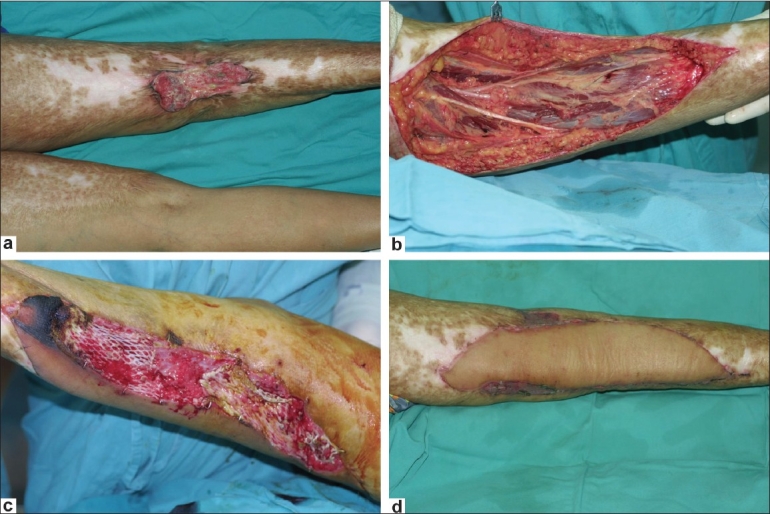
(a) A pre-operative photo of Marjolin.s ulcer at the right popliteal fossa; (b) An intra-operative photo after tumor excision with exposed neurovascular structures; (c) Two weeks post operation day; (d) Two weeks post secondary suturing of the wound

A 56-year-old female presented with Marjolin's ulcer at the back of her right knee secondary to a chronically non healing burn wound [Figure 1a]. A wide resection was done and a free latissimus dorsi myocutaneous free flap was required to cover the defect due to exposed neurovascular bundles [Figure 1b] and post-operative radiotherapy was planned. Unfortunately, due to prolonged surgery and ensuing edema, direct closure of the wound was not possible as it would have compressed the flap pedicle and jeopardized the flap perfusion. Therefore, the GPA was used to allow the edema to settle and anastomosis to become stable before the wound could be secondarily sutured after 2 weeks. By this time, the skin allograft had partially sloughed off, the wound bed was healthy and filled with granulation tissue, and there was wrinkling of the surrounding skin indicating resolved edema [Figure 1c]. This provided a better aesthetic outcome and avoidance of split skin graft to provide more robust and stable tissue for radiotherapy, and a better scar across the knee joint [Figure 1d].

### Case 2

**Figure 2:**
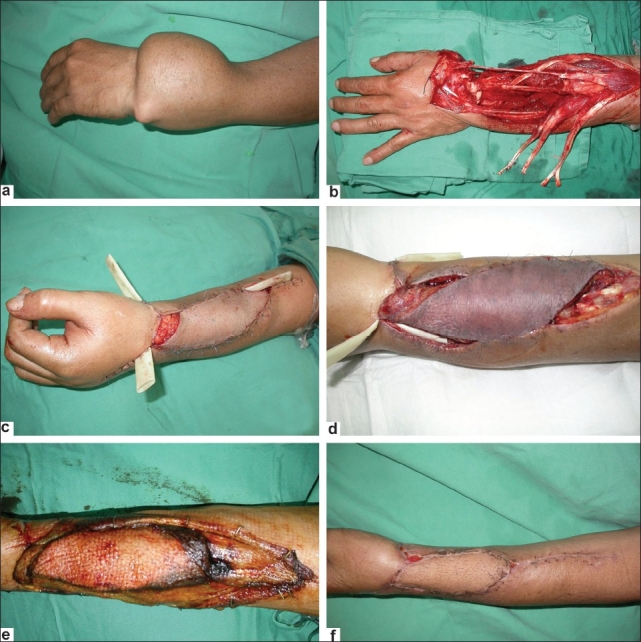
(a) Pre-operative giant cell tumor of left distal radius and carpal bones; (b) Preoperative following resection of the tumor; (c) Immediate postoperative with flap insetted; (d) Forty-eight hours post operative-flap congested due to venous thrombosis; (e) Two weeks after emergency exploration with GPA in situ and well adhered, and evidence of resolved edema with surrounding skin wrinkles; (f) One week after secondary suturing

A 43-year-old male presented with a massive giant cell tumor involving the distal end of the left radius and the first row of the carpal bones [Figure 2a]. A wide resection of the tumor was performed [Figure 2b] followed by reconstruction with a free fibula osteocutaneous flap. Fusion of the wrist joint was performed in the same setting [Figure 2c]. Twenty-four hours later, venous congestion was noted secondary to pedicle compression from local edema, which developed post operatively despite limb elevation [Figure 2d]. Emergency exploration was done and venous thrombosis was noted. This required a vein graft to re-establish venous outflow. Tension-free closure of the wound was not possible at the time due to severe edema. A GPA was used as a biological dressing for 10 days to allow the edema to subside and to protect the exposed tendon from desiccation [Figure 2e]. Secondary suturing was done at that stage, which provided a better aesthetic outcome compared with a split skin graft if it had been done at the time of flap exploration [Figure 2f].

### Case 3

**Figure 3:**
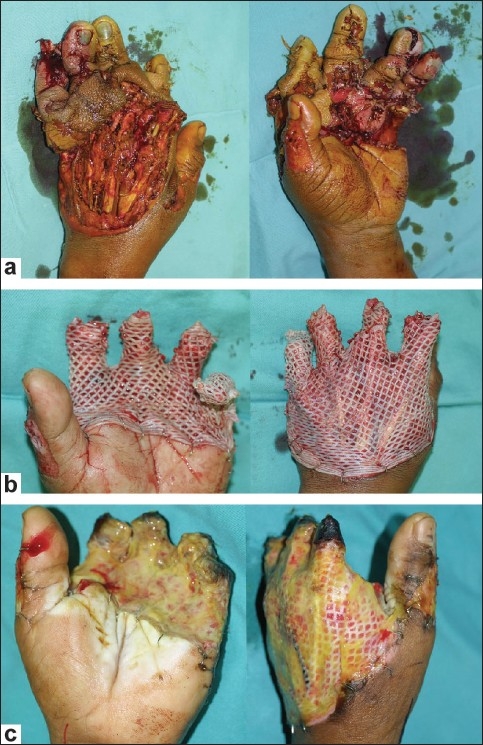
(a) Left hand crush injury due to sugarcane juicer machine; (b) GPA applied to the wound after first debridement; (c) Ten days after debridement with GPA well adhered and small patchy area of devitalized tissue becomes apparent

**Figure 4:**
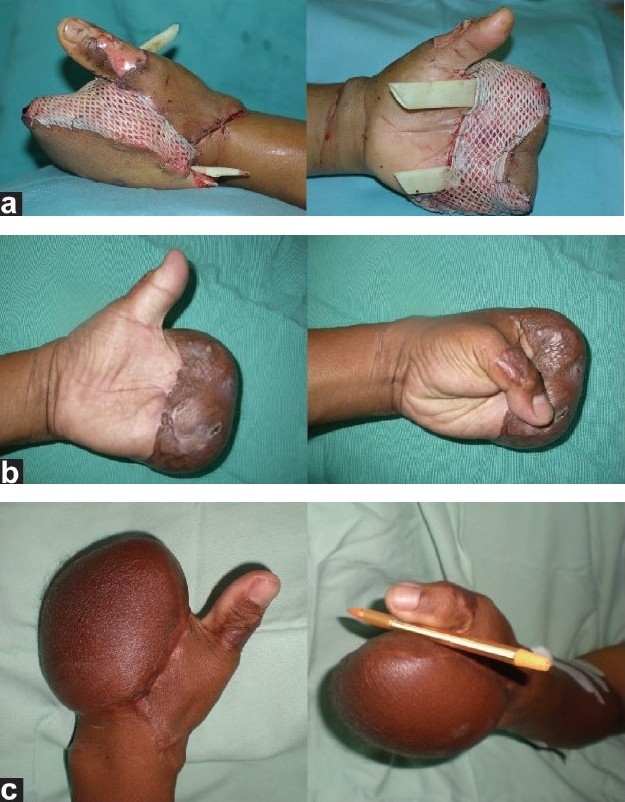
Left hand crush injury after debridement and pretreatment with GPA; (a). Immediate post-operative view after reconstruction with anterolateral thigh fasciocutaneous free flap, partial closure, and GPA application; (b) and (c). Three months and 1 year after secondary suturing of the flap

A 36-year-old male sustained a severe crush and degloving injury to his left hand with a sugar cane juicer machine. Most of his fingers were non-viable at presentation with bone loss [Figure 3a]. A conservative debridement was done and multiple vital structures such as bone, tendons, nerves, and vessels were exposed. All viable tissues were preserved and temporarily covered with a GPA [Figures 3b and 3c] and vacuum assisted closure dressing (VAC^®^). Several days later, the wound was reassessed and further debridement was done; an anterolateral thigh free flap was used to cover the wound. A GPA was needed to aid in wound closure without tension [Figure 4a]. A definitive wound cover with split skin graft was done 3 weeks later. The patient recovered well but refused a secondary reconstruction as he had adapted well and had resumed work [Figures 4b and 4c].

## DISCUSSION

A GPA can be used as a temporary biological dressing for complex and complicated wounds that cannot be closed immediately. A GPA will protect and preserve the viable tissues from desiccation and necrosis, while allowing time for the edema and swelling to subside and for the anastomosis to become stable and less thrombogenic so that it can withstand more pressure and compression, permitting wound approximation in the case of free tissue transfer. The use of GPA allows the assessment of wound bed readiness to receive a skin autograft i.e., it can be used as a ‘take test’ for skin graft (well adherence and bleeding upon its removal).[12] One of GPA's peculiar properties compared with other skin allograft preparations is its ability to promote neovascularisation. Although the mechanism is still not well understood, the wound bed is better prepared with the use of a GPA.[2–5] For complex wounds where tissue viability is in question, a GPA can be used as a biological and temporary dressing until the non viable tissues are well demarcated, while preserving the vital structures and healthy tissues. Serial assessments during wound inspection can be done. Necessary intervention is possible at any time while the wound bed is covered and protected by the skin allograft.

Usually, skin allografts are used for burn patients for temporary wound cover after debridement of burn wounds, full-thickness burns after epifascial necrectomies, overlay of widely spread skin autografts, and also as a vascularised dermal replacement. There are only a few articles cited in literature mentioning the use of GPA beyond the scope of burn management.[2,6,7]

Major reconstructive surgery for oncological resection and traumatic injury can sometimes be prolonged and may be associated with tissue swelling and edema. This can prevent tension-free wound closure and may require split thickness skin graft to cover the raw areas. The use of GPA as illustrated in our cases provides a means of wound cover until the swelling has settled and the wound edges can be secondarily sutured or can be partially closed with a skin graft. Several advantages are gained by using GPA in this setting; it can ensure a complete take of a skin graft with better wound bed preparation, it minimizes the use of skin grafts, and it gives better aesthetic outcome. These were well illustrated in all of our cases.

A GPA was used in these cases due to its advantages as a biological dressing, comparatively lower cost, ease of storage, and easy availability compared with the other skin allograft preparations. It is also more robust than the other biological dressings available, such as amnion and collagen. From our experiences, in these sort of cases, one can expect a lot of oozing and fluid discharges from the wound bed or the surrounding area, which requires regular dressing changes. A GPA could withstand the moist environment better than amnion or collagen and could tolerate regular handling without rapid deterioration of its physical and biological properties.

In the first case [Figure 1], direct closure of the wound was possible in the first instance but this had caused considerable tension on the skin paddle and subsequently, compression of the pedicle. We undertook a conservative approach by leaving one half of the flap edge open, covered with a skin allograft until the edema subsided and vascular anastomosis became stable, prior to secondarily closing the wound at the second sitting. Other alternatives would be using the SSG or dressing material to cover the wound.

A GPA can also be used in emergency situations as depicted in our second case [Figure 2] when the ensuing edema had caused venous compression and later thrombosis, needing vein graft interposition. A GPA was used to cover the wound until the flap pedicles across the anastamosis become stable and the edema subsided before secondary closure was possible after 2 weeks.

In the last illustrated case [Figure 3], the GPA was used twice in the patient who had sustained a severe, mutilated hand injury caused by a sugar cane juicer machine. He underwent emergency exploration and wound debridement after the injured digits were deemed unsalvageable. However, conservative debridement and preservation of the length is vital in managing hand injuries to give the best possible outcome; and the use of a GPA in this setting allowed conservative debridement. It can even be used on bare bones, tendons, and nerves preventing further deteriorating of these structures, as is evident in this case. Serial assessments are made to inspect for devitalized tissues that can be debrided prior to final reconstruction. As seen in the two earlier cases, the GPA was used in conjunction with a free tissue transfer to provide a tension free wound cover to protect the flap pedicle from compression. Another benefit of GPA in this case was preparing the wound bed for SSG. As we know, a GPA can promote neovascularisation of the wound bed and can be used as a ‘take test’ for the readiness of the wound bed to receive a skin autograft.[12]

Usually, the GPA can be left *in situ* for about 8 days before it will show signs of rejection.[13,14] In the first two cases in our series, the GPA was applied for 11 and 10 days, respectively, and it was found well adhered at the time of removal. In the last case, the first application lasted for 10 days with complete allograft adherence. In the second application, the GPA was left for 18 days with more than 60% of the allograft still adhering well. In these cases, the GPA seemed to adhere longer than previous data has shown. We have postulated that due to the small amount of GPA used, the inflammatory reaction inflicted in our cases is lower than that in previous literature data, from major burns studies.[13,14]

## CONCLUSIONS

GPA can be used in conjunction with free flap reconstruction to allow tension-free wound closure, thus protecting the flap pedicle. It can also be used to cover raw areas if any, until edema associated with the injury and surgery subsides. Later, this may allow secondary wound closure or partial closure with minimal amount of autograft used for better aesthetic outcome.
